# Multidimensional psychosocial characteristics of adolescents with idiopathic scoliosis in China: a controlled cross-sectional study

**DOI:** 10.1186/s40359-026-04470-0

**Published:** 2026-04-07

**Authors:** Huanjie Huang, Guifang Zhang, Xiaoqian Hu, Zhenfa Zhang, Haoyu Xie, Chuhuai Wang

**Affiliations:** 1 Department of Rehabilitation Medicine, The First Affiliated Hospital of Sun Yat-Sen University, Guangzhou, Guangdong China; 2 Guangdong Engineering and Technology Research Centre of Rehabilitation Medicine and Translation, Guangzhou, Guangdong China; 3 Department of Rehabilitation Medicine, Suzhou Municipal Hospital, Suzhou Rehabilitation Hospital, Gusu School, The Affiliated Suzhou Hospital of Nanjing Medical University, Nanjing Medical University, Suzhou, Jiangsu China

**Keywords:** Adolescent idiopathic scoliosis, Anxiety, Mental status, Psychosocial behavior

## Abstract

**Background:**

Adolescent idiopathic scoliosis (AIS) is the most common musculoskeletal disorder affecting adolescents. Despite its multifaceted implications, healthcare professionals often prioritise correcting spinal deformities over addressing the psychological ramifications. Our study aimed to comprehensively examine the psychosocial characteristics and mental health outcomes of adolescents with AIS in China.

**Methods:**

We recruited 61 participants with AIS and 61 healthy controls aged 10–18 years. Mental health status, psychosocial behaviour, and health-related quality of life (HRQoL) were evaluated using self-reported versions of the Strengths and Difficulties Questionnaire (SDQ-S), Self-rating Anxiety Scale (SAS), nine-item Patient Health Questionnaire (PHQ-9), and Scoliosis Research Society-22 Questionnaire (SRS-22). The Shapiro-Wilk normality test and Levene’s test were used to measure the normality and homogeneity of variance of each dependent variable, respectively. For *p*-values of > 0.05, an independent-sample t-test was used to identify any significant differences between participants with AIS and healthy controls. For *p*-values of < 0.05, the Mann-Whitney U-test was performed. The chi-square test was used to compare differences in dichotomous variables. Pearson and Spearman correlation analyses were used to test the correlations between continuous and ordinal categorical variables. Statistical significance was set at *p*-values of < 0.05.

**Results:**

The psychosocial behaviour and mental status-related domains of SRS-22 and SDQ-S differed significantly between adolescents with AIS and healthy controls in China. The SAS results showed that AIS participants had notably higher anxiety levels compared to controls. The Cobb angle significantly correlated with the function domain scores, SRS-22 scores, and anxiety levels of adolescents with AIS in China.

**Conclusions:**

AIS impairs the mental status and psychosocial behaviour of adolescents, significantly reducing their HRQoL. Regardless of scoliosis type, a large Cobb angle leads to increased anxiety in adolescents with AIS rather than depression. This highlights that early mental health evaluations and interventions for Chinese adolescents with AIS are crucial.

**Trial registration:**

This observational study was registered in the Chinese Clinical Trial Registry (https://www.chictr.org.cn/) on August 12, 2024, under the registration number ChiCTR2400088100.

## Background

Adolescent idiopathic scoliosis (AIS) is the most common form of structural spinal deformity in adolescents, typically occurring between 10 and 18 years of age [1, 2]. Large-scale screening studies in China have reported that the overall prevalence of AIS is approximately 2–3% among school-aged children and adolescents [3, 4]. Although AIS is primarily characterised by a three-dimensional spinal deformity, increasing evidence suggests that its impact extends beyond physical appearance and may affect psychological well-being and social functioning [5]. Previous studies have suggested that, in addition to physical deformity, AIS may also adversely influence adolescents’ psychological health. Adolescents with AIS experience a deterioration in psychological health associated with a negative attitude towards life, low self-esteem, and difficulties in social connections and peer relationships [5]. While acknowledging the multifaceted implications of AIS, healthcare professionals often prioritise correcting spinal deformities over addressing the psychological ramifications, as reflected in the existing literature [6, 7]. Given the high incidence of mild-to-moderate AIS, it is imperative to address the psychological health of this subpopulation, as this is integral to the overall prognosis of the condition. Therefore, a more comprehensive understanding of the psychosocial characteristics of adolescents with AIS is therefore essential for improving holistic management strategies.

Anxiety and depression are increasingly prevalent among Chinese adolescents, posing serious challenges to their mental health and overall development [8]. Adolescents with AIS may be particularly susceptible to such psychological problems. Clinically significant psychosocial and emotional problems are observed in 32% of patients with AIS [5]. Previous study has reported that adolescents with AIS may experience various psychosocial difficulties, including anxiety, depressive symptoms, body image disturbance, and problems in peer relationships [7]. Adolescents with AIS may experience significant psychosocial problems, including anxiety, depression, and social maladjustment, which can negatively affect their mental health and quality of life [9, 10]. A series of clinical assessments and questionnaires are commonly used to evaluate the psychosocial behaviour and mental status of patients with AIS. The Strengths and Difficulties Questionnaire (SDQ) is a widely used generic screening instrument for assessing psychosocial well-being in children and adolescents, and has been applied in previous studies, including those involving adolescents with AIS, to evaluate mental health perceptions from both patient and parent perspectives [11, 12], according to five subscales. AIS causes poor psychological outcomes (emotional symptoms) and various psychosocial abnormalities (difficulties in peer relationships or prosocial behaviour) in adolescents [11]. These psychological issues may vary among patients with AIS, and a comprehensive assessment is therefore necessary. Furthermore, anxiety and depression, the most prevalent emotional disorders in adolescents, should be given sufficient attention [13]. The Self-Rating Anxiety Scale (SAS) and nine-item Patient Health Questionnaire (PHQ-9) are widely used clinical tools to assess anxiety and depression [14]. However, studies simultaneously assessing multiple psychosocial dimensions-including general psychological difficulties, anxiety, depression, and health-related quality of life-in adolescents with AIS remain relatively limited. Based on available evidence, AIS is a risk factor for mental disorders, and patients with AIS have a higher probability of developing depression or anxiety [15]. These psychological problems result in decreased health-related quality of life (HRQoL), referring to an individual’s level of happiness or satisfaction with their personal life in the face of disease or treatment [16, 17]. The Scoliosis Research Society-22 Questionnaire (SRS-22) is widely used to specifically assess the HRQoL of patients with AIS [9, 18–20]. According to the SRS-22, patients experience lower HRQoL to different extents because of physical and mental impairments due to scoliosis [9, 18].

Some researchers have suggested that the level of mental stress experienced by an individual with AIS depends on the Cobb angle; a more severe deformity will cause greater psychological distress and a higher risk of depression [21]. However, recent evidence suggests that psychological distress may occur even in the presence of relatively mild scoliosis, indicating that the mere presence of scoliosis may affect mental health [13]. Therefore, this study aimed to investigate how AIS affects the psychosocial behaviour and mental status of patients in China. We hypothesised that patients with AIS have a higher risk of psychological distress and lower HRQoL than healthy adolescents and that the Cobb angle is inversely correlated with mental health and HRQoL.

## Methods

### Participants

This observational cross-sectional study recruited adolescents with AIS and healthy controls aged 10–18 years between September 2022 and September 2023.

Inclusion criteria for the AIS group were: (1) diagnosis of adolescent idiopathic scoliosis according to the 2016 SOSORT guidelines [2] ; (2) Cobb angle between 10° and 40°; (3) age between 10 and 18 years; and (4) ability to complete self-reported questionnaires independently.

Exclusion criteria were: (1) scoliosis secondary to neuromuscular, congenital, or syndromic conditions; (2) spondylolysis; (3) history of spinal surgery or spinal trauma; (4) Cobb angle > 40°; and (5) presence of other neurological or psychiatric disorders.

Healthy controls were recruited from local schools and community settings during the same period and were matched to the AIS group by age and sex.

Inclusion criteria for healthy controls included: (1) absence of a prior diagnosis of scoliosis or other spinal deformities, as confirmed by self-report and available school health records; (2) no history of musculoskeletal, neurological, or psychiatric disorders; and (3) ability to complete self-reported questionnaires independently.

On the day of data collection, full-length standing posterior–anterior and lateral radiographs were obtained only from participants with AIS. All participants and their guardians were instructed to complete a series of questionnaires.

The sample size was determined based on a previous publication [5] via the computation of power analysis using G*power (http://www.gpower.hhu.de/); the recruitment of 92 participants generated a power of 80% and a significance level of 5% (two-sided) to detect true differences in psychological distress as measured by the total difficulties score of the SDQ [5]. According to the partial Eta-squared method, η^2^ = 0.138 (derived from between-group differences in SDQ total scores) as the large effect size was used to calculate the effect size *f* [22]. Considering a calculated effect size *f* of 0.4, recruiting 61 patients with AIS and 61 healthy controls was statistically sufficient to identify the true differences in psychosocial behaviour and mental status. This study received ethical approval from the Institutional Ethics Committee of the First Affiliated Hospital of Sun Yat-sen University (IRB# [2022] 479) and was registered in the Chinese Clinical Trial Registry (https://www.chictr.org.cn/), under the registration number ChiCTR2400088100. Written informed consent was obtained from each participant and their respective legal guardian on the day of data collection. Detailed information about this study was also provided.

### Outcome measures and clinical variables

The primary outcome measures of this study were psychosocial behaviour, mental health status, and HRQoL, assessed using the self-reported SDQ-S, SAS, PHQ-9, and SRS-22, respectively.

The Cobb angle and scoliosis curve pattern (single or double curve) were recorded as clinical characteristics of participants with AIS and were used as explanatory variables in correlation analyses, rather than as outcome measures.

The Cobb angle and scoliosis type on the posterior–anterior radiographs were evaluated by two experienced physical therapists. The Cobb angle was independently measured by both raters, and the average value was used for participants with AIS. Any discrepancies between raters were reviewed and resolved by consensus. Although formal inter-rater reliability statistics were not calculated, differences between raters were small and considered clinically negligible.

To evaluate HRQoL, the SRS-22 was administered to all participants. SRS-22 has five domains (function, pain, mental health, self-image, and satisfaction with treatment) with 22 items and demonstrates good reliability and validity for assessing HRQoL specific to scoliosis [23]. Each item scored 0–5, with a higher score representing a better HRQoL [2]. The satisfaction with treatment domain of the SRS-22 was analysed descriptively in participants with AIS only and was not used for comparisons with healthy controls.

Self-reported versions of the SDQ (SDQ-S), SAS, and PHQ-9 were used to evaluate the psychosocial behaviour and mental status of participants with AIS and healthy controls. SDQ-S is a brief emotional and behavioural screening questionnaire for children and adolescents used to capture their perspective [24]. SDQ-S has five subscales with a total of 25 items (emotional symptoms, conduct problems, hyperactivity/inattention, peer relationship problems, and prosocial behaviour) [25]. Prosocial behaviour involves voluntarily acting in ways that conform to social expectations, offering benefits to others—such as helping, sharing, cooperating, comforting, or donating-without obvious personal gain [25]. Higher SDQ-S scores indicate more severe psychosocial issues [25]. SAS is a norm-referenced scale developed by Zung in 1971 that covers a variety of anxiety symptoms and demonstrates satisfactory psychometric properties [26]. SAS includes 20 items, scoring 1–4. A higher score indicates a higher level of anxiety. The SAS cut-off score was 40 (40–59: mild-to-moderate; 60–74: moderate-to-severe; 75–80: extreme) [14]. The PHQ-9 consists of nine self-reported items covering the Diagnostic and Statistical Manual of Mental Disorders, Fifth Edition, diagnostic criteria for major depressive disorders [27]. The PHQ-9 was used to evaluate the frequency of depressive symptoms in the past 2 weeks, with a higher score indicating a greater level of depression [27].

Participants and their guardians were instructed to complete a series of questionnaires. All questionnaires were completed independently by the participants in a quiet setting. A trained researcher was present only to provide standardized instructions and to clarify procedural questions if necessary, without offering any interpretation or guidance related to questionnaire content. Guardians were not involved in answering the adolescents’ self-reported questionnaires.

This study was designed as a cross-sectional observational study focusing on the psychosocial and mental health characteristics of adolescents with AIS, rather than on the effects of specific treatments. Therefore, treatment-related variables such as bracing duration, physiotherapeutic scoliosis-specific exercises (PSSE), and years of treatment were not included in the analysis.

### Statistical analysis

Statistical analyses were performed using SPSS (version 20.0; IBM Corporation, Armonk, NY, USA). Continuous variables were expressed as mean ± standard deviation for normally distributed data and as median (interquartile range) for non-normally distributed data. Given that the SRS-22 domain and total scores are derived from Likert-scale items, their distributions were examined using the Shapiro–Wilk test, and appropriate parametric or non-parametric tests were applied accordingly. The Shapiro–Wilk normality test and Levene’s test were used to measure the normality and homogeneity of variance of each dependent variable, respectively. Between-group comparisons were conducted using independent-sample t tests for normally distributed data with homogeneous variances, and Mann–Whitney U tests for non-normally distributed data. The chi-square test was used to compare differences in dichotomous variables. Pearson and Spearman correlation analyses were used to test the correlations between continuous and ordinal categorical variables. Statistical significance was set at *p*-values of < 0.05.

## Results

### Characteristics of participants

#### Participant flow

A total of 74 adolescents with suspected scoliosis were initially assessed for eligibility. Of these, 13 were excluded due to Cobb angle > 40°, presence of secondary scoliosis, or incomplete questionnaire data. Finally, 61 adolescents with AIS were included in the analysis.

During the same period, 61 healthy adolescents who met the inclusion criteria were recruited as controls and matched to the AIS group by age and sex.

No statistically significant differences in baseline characteristics were observed between participants with AIS and healthy controls (*p* > 0.05; Table 1). The average Cobb angle of participants with AIS was 20.39 ± 8.93°. A single major curve (C-shaped) was identified in 33 participants with AIS, with the major curve located in the thoracic or thoracolumbar/lumbar region, and the average Cobb angle was 23.79 ± 8.77°. Double major curves (S-shaped) were observed in 28 participants, and the Cobb angle used for analysis represented the larger (more severe) curve rather than the average of both curves, with an average value of 17.52 ± 8.12°.

**Table 1:** Demographic characteristics of AIS patients and healthy controls

	Participants with AIS (*n* = 61)	Healthy controls(*n* = 61)	*p*-value
Sex (male/female)	18/43	18/43	1.000
Age (years)	15.55 ± 2.53	15.52 ± 2.72	0.522
Height (cm)	159.05 ± 9.60	158.71 ± 9.63	0.104
Weight (kg)	46.30 ± 8.05	48.05 ± 11.10	0.137
Body mass index (kg/m2)	18.27 ± 2.66	18.83 ± 2.92	0.495
Degree of education (# of participants)			
Primary school	18 (29.5%)	16 (26.2%)	0.211
Middle school	30 (49.2%)	28 (45.9%)	
High school or higher	13 (21.3%)	17 (27.9%)	
Family history of scoliosis	2 (2.4%)	1 (1.2%)	0.658
Cobb’s angle (°)	20.39 ± 8.93	/	/
Shape of scoliosis (single/double curve, %)	54.1%/45.9%	/	/

Data were presented as mean±standard deviation

### Impact of Cobb angle on locomotor function and HRQoL in participants with AIS

Healthy controls were not involved in the AIS treatment; thus, satisfaction with the treatment domain in the SRS-22 scored 10.

**Fig. 1:**
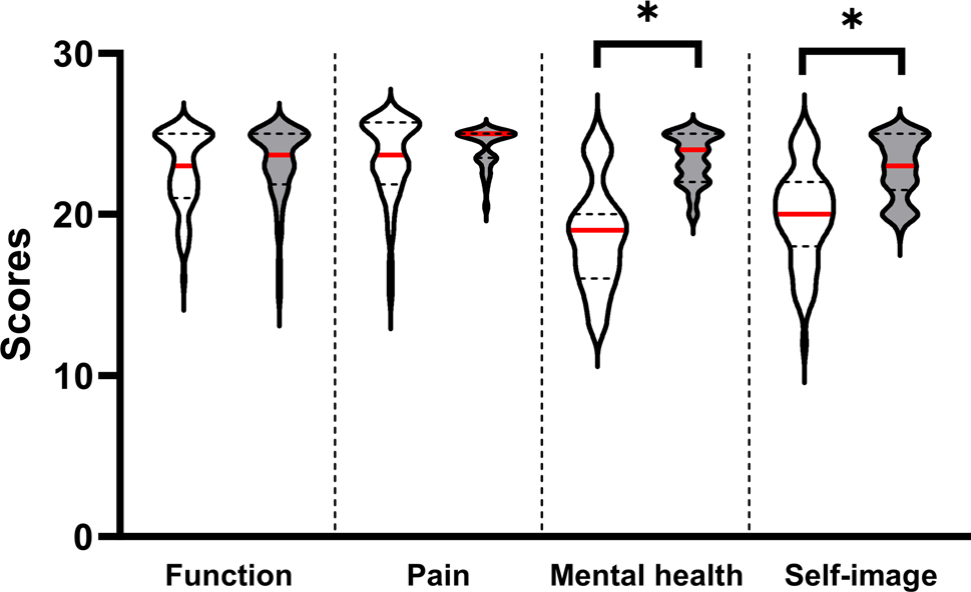
Violin plot for five domain scores from the SRS-22 of participants with AIS and healthy adolescents. Participants with AIS are shown as a white-fill pattern; healthy adolescents are shown as a grey-fill pattern. The red line represents the median (50%); dash lines represent quartiles (25% and 75%). * indicates the significant difference between participants with AIS and healthy adolescents. The satisfaction with treatment domain was analysed descriptively in the AIS group only and was excluded from between-group comparisons. Abbreviations: SRS-22, Scoliosis Research Society-22; AIS, adolescent idiopathic scoliosis

**Fig. 2:**
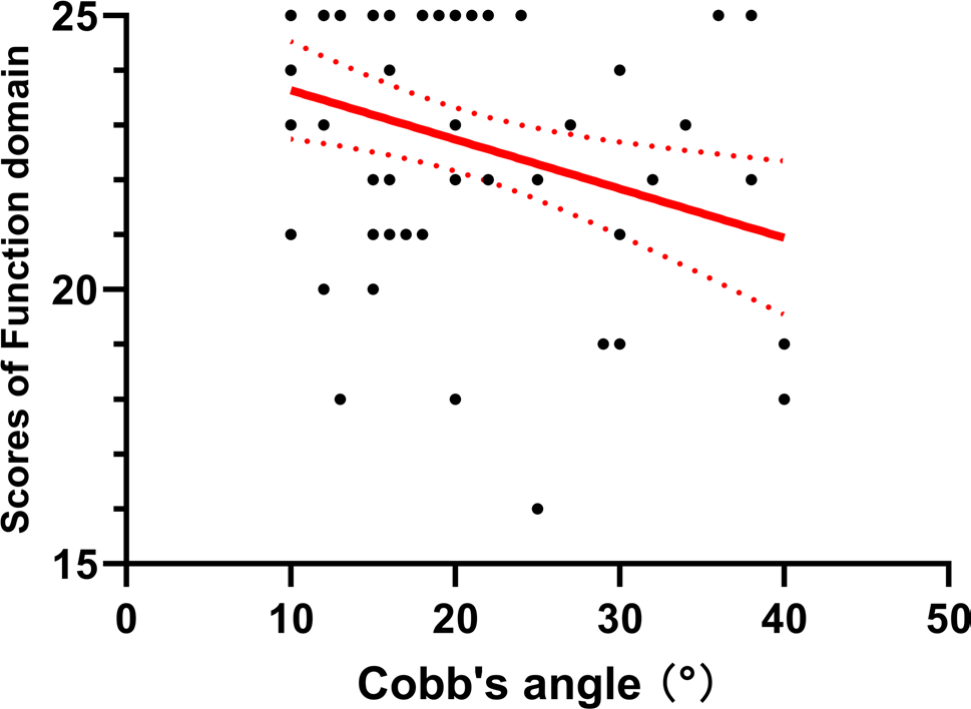
Scatter plot between Cobb’s angles and Function domain scores from SRS-22 for participants with AIS. The red line represents the regression line; the red dash lines represent the 95% confidence band. Abbreviations: SRS-22, Scoliosis Research Society-22; AIS, adolescent idiopathic scoliosis

**Fig. 3:**
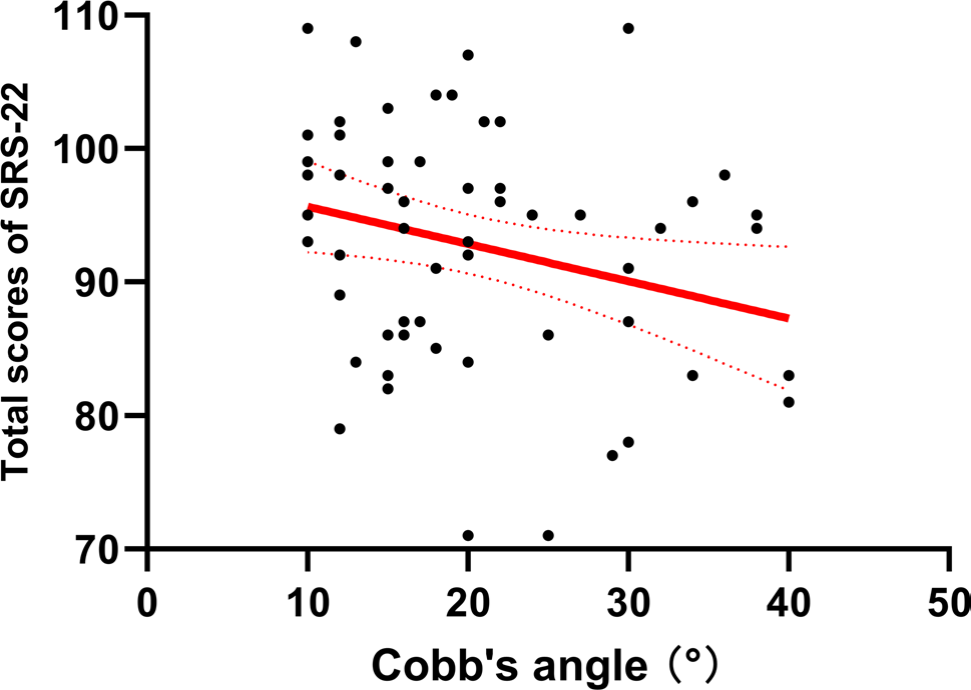
Scatter plot between Cobb’s angles and total scores of the SRS-22 for participants with AIS. The red line represents the regression line; the red dash lines represent the 95% confidence band. Abbreviations: SRS-22, Scoliosis Research Society-22; AIS, adolescent idiopathic scoliosis

Participants with AIS demonstrated significantly lower mental health (*p* = 0.008) and self-image (*p* = 0.012), as well as lower total SRS-22 scores (*p* = 0.006), compared with healthy controls (Fig. 1). The satisfaction with treatment domain of the SRS-22 was not included in the between-group comparison, as this domain is applicable only to patients who have received treatment. In participants with AIS, no significant differences were observed between single and double curves in each domain (*p* > 0.05). Pearson’s correlation analysis showed that the Cobb angle was significantly correlated with the functional domain (*r*=-0.338, *p* = 0.008) and the total score (*r*=-0.280, *p* = 0.029) of the SRS-22, indicating that a greater Cobb angle led to poorer locomotor function and HRQoL in participants with AIS (Figs. 2 and 3).

### Impaired peer relationships and prosocial behaviour in participants with AIS

**Fig. 4:**
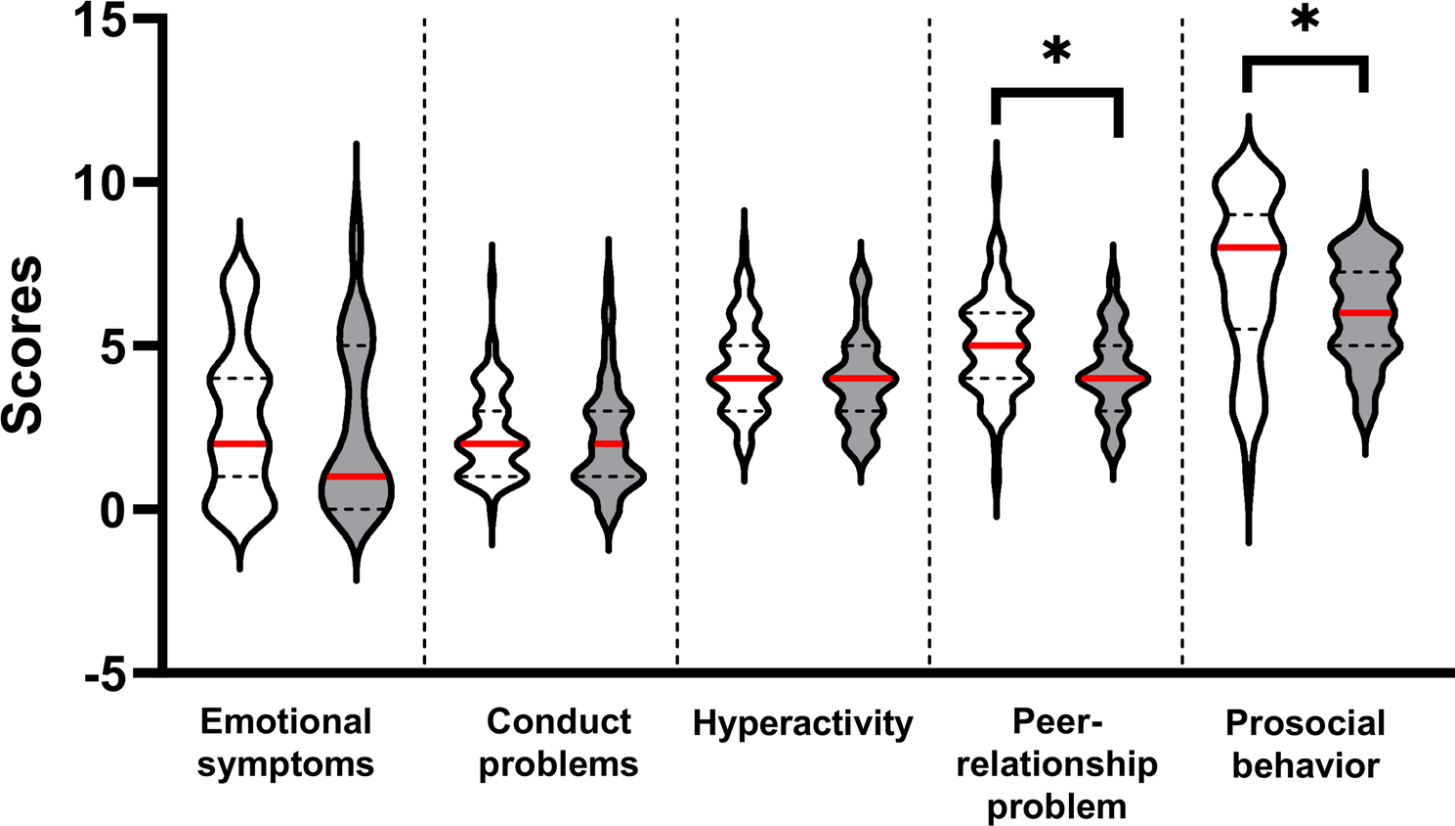
Violin plot for five subscale scores from SDQ-S for participants with AIS and healthy adolescents. Participants with AIS are shown as a white-fill pattern; healthy adolescents are shown as a grey-fill pattern. The red line represents the median (50%); dash lines represent quartiles (25% and 75%). * indicates the significant difference between participants with AIS and healthy adolescents. Abbreviations: SDQ-S, self-reported version of the Strengths and Difficulties Questionnaire; AIS, adolescent idiopathic scoliosis

Participants with AIS and healthy controls showed significant differences in the subscales of peer relationship problems (*p* = 0.004) and prosocial behaviour (*p* = 0.023), as well as in the total scores of SDQ-S (*p* = 0.010; Fig. 4). No significant differences were observed in any subscale or the total SDQ-S score between participants with single and double curves (*p* > 0.05). Pearson’s correlation analysis revealed no significant correlation between the Cobb angle and each subscale or the total SDQ-S score (*p* > 0.05).

### Association between Cobb angle and anxiety, but not depression, in participants with AIS

**Fig. 5:**
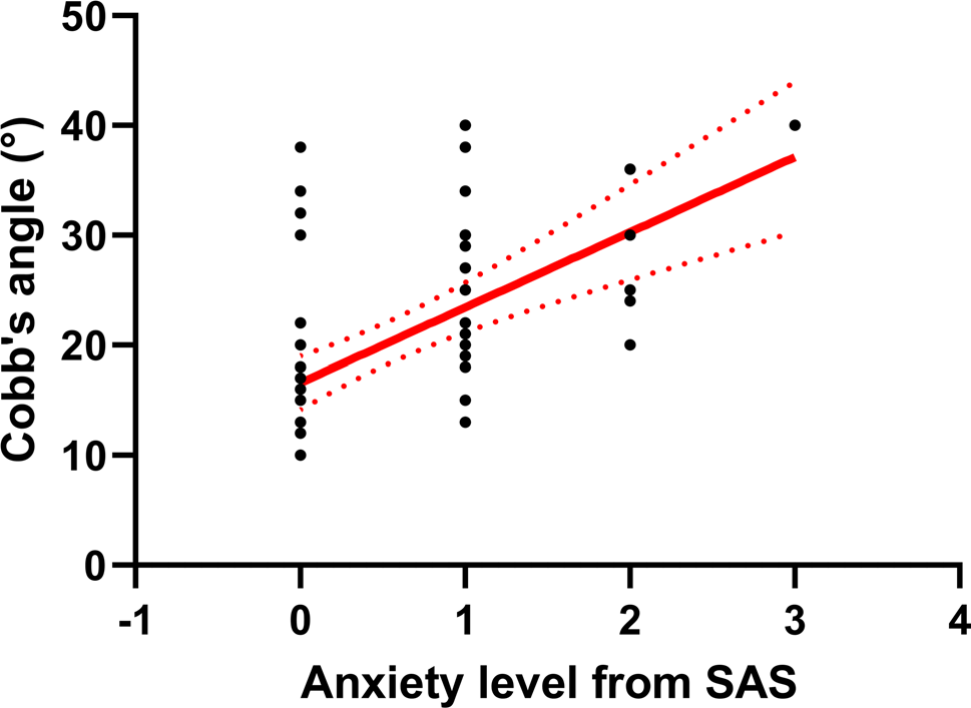
Scatter plot between Cobb’s angles and anxiety level from the SAS for participants with AIS. The red line represents the regression line; the red dash lines represent the 95% confidence band. Abbreviations: SAS, Self-rating Anxiety Scale; AIS, adolescent idiopathic scoliosis

Participants with AIS had significantly higher anxiety level than healthy controls (Z=-3.547, *p* < 0.001) according to SAS, but no significant difference was observed in depression levels on the PHQ-9 (Z=-0.212, *p* = 0.832; Table 2). For participants with AIS, no significant differences were observed in anxiety (Z=-0.624, *p* = 0.532) and depression (Z=-0.094, *p* = 0.925) levels between those with single and double curves. Spearman correlation analysis suggested that the Cobb angle was significantly correlated with anxiety (ρ = 0.601, *p* = 0.005) but not depression (ρ = 0.237, *p* = 0.066), indicating that a greater Cobb angle led to greater anxiety (Fig. 5).

**Table 2:** Anxiety and depression levels in AIS patients and healthy adolescents (SAS and PHQ-9)

	Participants with AIS (*n* = 61)	Healthy controls (*n* = 61)	Z-value	*p*-value
SAS				
Normal	34 (55.7%)	54 (88.5%)	-3.547	< 0.001*
Mild to moderate	21 (34.4%)	7 (11.5%)		
Moderate to severe	5 (8.3%)	0 (0%)		
Extreme	1 (1.6%)	0 (0%)		
PHQ-9				
Normal	29 (47.5%)	31 (50.8%)	-0.212	0.832
Mild	20 (32.8%)	17 (27.9%)		
Moderate	7 (11.5%)	12 (19.7%)		
Severe	5 (8.2%)	1 (1.6%)		

*Abbreviations*: *SAS* Self-rating Anxiety Scale, *PHQ-9* Nine-item Patient Health Questionnaire

## Discussion

This study revealed that adolescents with AIS had impaired mental health status and psychosocial behaviour, as well as lower HRQoL compared to healthy controls. Furthermore, patients with AIS exhibited higher levels of anxiety, which was significantly positively correlated with the Cobb angle.

Although anxiety and depression are highly comorbid and share common aetiological processes, differences in their clinical manifestations exist between the two psychological conditions [28]. Depression is a mood disorder that causes a persistent feeling of sadness and loss of interest that interferes with daily activities, whereas anxiety is an emotional state characterised by feelings of tension and intrusive thoughts [29]. Anxiety and depression play important roles in mental health; however, previous studies have suggested that anxiety (15–20%) is a more prevalent psychological condition than depression (3–15%) in children with AIS [5, 28]. Our results support the finding that AIS significantly increases the risk of anxiety but not depression in adolescents.

Three potential sources of increased anxiety in patients with AIS were identified. Firstly, anxiety may arise from fear and the unknown nature of AIS. Adolescents with limited medical knowledge often struggle to understand scoliosis correctly, leading to misconceptions that can affect their mood and behaviour [21]. Additionally, because AIS may progress continuously, patients usually experience a transition from scoliosis to chronic disease, causing greater anxiety [15]. Secondly, increased anxiety may be caused by social connections with peers. As peer relationships become increasingly important during adolescence, youngsters become more sensitive to the fear of being humiliated or scrutinised by peers [30]. While general models of adolescent social anxiety have been described previously [30], scoliosis-specific studies suggest that concerns related to spinal deformity and body image may uniquely contribute to anxiety in adolescents with AIS [31]. Adolescents often lack the psychological maturity to fully recognise and cope with the physical changes caused by scoliosis, particularly when these changes set them apart from their peers [15]. This causes social exclusion and peer rejection in adolescents with AIS, leading to greater social anxiety and lower self-esteem. Thirdly, exposure to parental anxiety has been associated with increased anxiety in offspring [32]. Previous studies have shown that parental anxiety affects the psychological condition of children with illnesses [5]. According to Hines et al. [33], parents of children diagnosed with AIS experienced elevated levels of anxiety, whereas those with children not diagnosed with AIS demonstrated a significant decline in anxiety. Therefore, parental attitudes towards scoliosis play an important role in maintaining good mental health in children with AIS, and family education and psychological counselling are helpful.

No significant difference in the depression level was observed between adolescents with AIS and healthy controls, suggesting that AIS was not associated with an increased risk of depression compared with peers without scoliosis. In contrast to previous studies, our results suggest that these differences may arise from differences in cultural backgrounds [15]. Available research has shown that depression is the most prevalent mental disorder among adolescents in North America [34] and Europe [35]. In contrast, some studies conducted in China have suggested that anxiety symptoms may be relatively more prominent among Chinese adolescents due to academic and social pressures [36]. However, the present study was not designed to evaluate the prevalence of mental health problems in the general adolescent population; therefore, these interpretations should be considered in the context of existing literature rather than as direct conclusions from our data. Stankov [35] showed that adolescents receiving Confucian education demonstrated greater anxiety and lower life satisfaction than European adolescents. Therefore, anxiety may be a more significant psychological condition than depression in Chinese adolescents with AIS. However, a substantial proportion of participants in both groups exhibited varying levels of depressive symptoms, highlighting that depression represents a non-negligible mental health concern during adolescence, regardless of the presence of AIS.

Our results suggest that greater Cobb angles are associated with higher levels of anxiety and poorer HRQoL, which is consistent with previous studies [5]. Previous research indicated that stress levels tend to rise with larger Cobb angles, but it also demonstrated that an increase in the Cobb angle did not result in greater difficulties in prosocial behaviour or peer relationships [9]. This suggests that the diagnosis of AIS itself may represent a primary psychosocial stressor, while disease severity may further exacerbate psychological burden rather than act as an isolated determinant [5]. Adolescents diagnosed with scoliosis may be concerned about the potential impact of AIS on their body image and social relationships. Hence, psychosocial difficulties may emerge early after diagnosis, even before scoliosis progresses to visibly affect physical appearance [5]. Additionally, no significant difference was found between the cases with single and double curves, suggesting that scoliosis type was not an important factor affecting the psychosocial functioning and mental health of patients with AIS. The observed associations between Cobb angle and anxiety or HRQoL should therefore be interpreted in the context of disease severity rather than treatment exposure. To our knowledge, few studies have specifically examined the psychological impact of different scoliosis curve types, and our findings contribute additional evidence in this area.

Several large-scale studies have reported no or only minimal psychosocial impact of adolescent idiopathic scoliosis or its conservative treatment, particularly in long-term follow-up cohorts. For example, population-based studies have shown that adolescents with mild to moderate scoliosis did not differ significantly from their peers in overall psychological well-being or social functioning in adulthood [37]. Similarly, some longitudinal studies suggested that bracing treatment, when appropriately indicated, may not result in clinically meaningful long-term psychological distress [38]. Conversely, other studies have reported significantly lower HRQoL among adolescents with AIS, particularly in domains related to self-image, emotional functioning, and social participation [17].

The discrepancies across studies may be explained by several factors. Differences in study design, population characteristics, severity of spinal curvature, treatment modalities, cultural background, and outcome assessment tools may all influence reported psychological outcomes. Additionally, many large cohort studies have focused primarily on long-term adult outcomes or global quality-of-life indicators, whereas the present study specifically examined anxiety and psychosocial functioning during adolescence. Adolescence is a developmental stage characterized by heightened sensitivity to body image and social evaluation, which may amplify the psychosocial impact of visible physical conditions such as scoliosis. Thus, the absence of long-term impairment reported in previous studies does not preclude the presence of short- or medium-term psychological burden during adolescence.

### Limitations

This study had some limitations. Firstly, the cross-sectional study enrolled only 122 participants. Nevertheless, based on the partial Eta-squared method, the effect size was moderate to large, and the study achieved 80% power, suggesting the practical significance of our findings. However, the relatively limited sample size and the size of the control group may still restrict the statistical robustness and generalizability of the findings. Future studies with larger samples and multi-centre recruitment are therefore warranted. Secondly, based on the inclusion criteria, patients with a Cobb angle > 40° were excluded. Hence, the psychosocial behaviour and mental health status of patients with severe AIS remain unknown. Our research team will attempt to answer these questions in future studies. Thirdly, treatment-related factors such as bracing, PSSE participation, and treatment duration were not analysed in this study. As the present research focused on the psychosocial impact of AIS itself and radiographic severity in a cross-sectional design, these treatment-related variables were not comprehensively controlled or stratified in the analysis. Future longitudinal studies are warranted to investigate the independent and combined effects of different treatment modalities on mental health outcomes. Fourthly, although the AIS and control groups were generally comparable in terms of basic demographic characteristics such as age, sex, and BMI, other potentially relevant factors—such as physical activity level and sports participation—were not systematically recorded or matched between groups. Physical activity may influence psychological well-being in adolescents and should therefore be considered in future studies. Finally, all participants were recruited from a Chinese population, which may limit the generalizability of the findings to adolescents from other cultural backgrounds. Cultural and social factors, including educational expectations, collectivist values, and culturally influenced patterns of emotional expression, may affect how adolescents perceive and report psychosocial distress. Therefore, caution is required when extrapolating the present results to non-Chinese populations.

## Conclusions

Chinese adolescents with AIS demonstrated significantly poorer psychosocial behaviour, mental health status, and lower HRQoL, compared to those of healthy adolescents. Increasing the Cobb angle was associated with greater anxiety and poorer HRQoL, whereas psychosocial functioning appeared to be impacted primarily by the presence of scoliosis rather than curve severity. Although depressive symptoms were observed in a substantial proportion of participants, no significant difference was found between adolescents with AIS and healthy controls, suggesting that depression represents a broader mental health concern during adolescence rather than an AIS-specific outcome. Therefore, healthcare professionals and parents should carefully considered mental health and HRQoL in adolescents with AIS to enable early assessments and appropriate psychological support. These findings also highlight the importance of multidisciplinary collaboration among physicians, physiotherapists, psychologists, and family members involved in the care of adolescents with AIS. Consistent communication, shared understanding, and aligned messages across the care team may facilitate functional management, enhance treatment adherence, and better support adolescents’ psychosocial well-being throughout the course of care. Future research should include larger cohorts, consider potential time-related differences and other correlating factors, aiming to improve the mental health of those affected by AIS.

## Data Availability

The data that support the findings of this study are available from the corresponding author upon reasonable request. Requests should be directed to Dr. Chuhuai Wang, wangchuh@mail.sysu.edu.cn.

## Abbreviations

AIS: Adolescent idiopathic scoliosis
HRQoL: Health-related quality of life
PHQ-9: Patient Health Questionnaire
SAS: Self-Rating Anxiety Scale
SDQ: Strengths and Difficulties Questionnaire
SRS-22: Scoliosis Research Society-22 Questionnaire
